# Supplementary material for: Are carbon emissions trading and green financial instruments synergistic? -Comprehensive quantitative research based on content analysis
Source: PLoS One. 2024 Mar 7;19(3):e0298601. doi: 10.1371/journal.pone.0298601 (PMC10919601; doi:10.1371/journal.pone.0298601)
Supplement: S1 Data — (ZIP) [file pone.0298601.s001.zip › Data/地级市Z_F工作报告.zip!/║■─╧/╥μ╤⌠╩╨/└┤╫╘╣1⁄2╓┌║┼í╛┬φ┐╦╩2╛▌═°í┐_2006.docx]

各位代表：

　　现在，我代表市人民政府，向大会作政府工作报告，请予审议，并请各位政协委员和其他列席人员提出意见。

　　一、“十五”计划执行情况和2005年工作回顾

　　 （一）“十五”计划主要指标完成情况

　　“十五”时期，在市委的正确领导下，全市人民认真贯彻执行党的路线方针政策，牢牢抓住发展这个党执政兴国的第一要务，聚精会神搞建设，一心一意谋发展，成功战胜各种自然灾害，国民经济持续快速发展，人民生活进一步改善，各项社会事业取得新进步，全面完成或超额完成了“十五”计划确定的经济社会发展各项目标任务。预计2005年生产总值达到329.2亿元，年均增长9.7%：人均生产总值达到8169元，年均增长9.8%。完成财政总收入15.24亿元，年均增长14.8%，其中地方财政收入9.24亿元，年均增长10.2%。实现工业增加值74.8亿元，年均增长11.9%；规模工业实现利润1.8亿元，比2000年增加2.2亿元，结束了长期亏损局面。农林牧渔业实现总产值130亿元，年均增长5.8%。”五年累计完成全社会固定资产投资346亿元，年均增长16%；累计实际利用外资2.9亿美元，年均增长21%，累计实际利用内资145亿元，年均增长61%；累计完成进出口总额4.2亿美元，年均增长45.7%。2005年，实现社会消费品零售总额116亿元，年均增长10.4%。农民人均纯收入达到2975元，年均增长8.6%；城镇居民人均可支配收入达到8250元，年均增长8.1%。全市城镇化率达到35%，比2000年提高5.8个百分点。

　　（二） 2005年工作回顾

　　 2005年，全市各级各部门认真贯彻落实“三个代表”重要思想，坚持以科学发展观统领全局，进一步突出加快发展主题，自加压力，开拓进取，国民经济和社会发展取得了新的成就。

　　经济发展速度加快。预计生产总值比上年增长11%，其中第一、二、三产业增加值分别增长6.4%、14.1%、11.2%。完成全社会固定资产投资100亿元，增长30.1%。社会消费品零售总额增长13.4%。财政总收入增长16.6%，同口径比增长25.7%：其中地方财政收入增长11.3%，同口径比增长25.6%。年末金融机构各项存款余额246.7亿元，各项贷款余额159.6亿元，比年初分别增长15.9%和15.5%。

　　产业建设力度加大。实施工业强市战略取得成效。预计工业增加值比上年增长13.3%，其中规模工业增加值增长22%，利润增长2倍，企业经济效益综合指数达到115.4%，比上年提高10个百分点．电力、食品加工、棉麻纺织、装备制造、造纸、建材等六大优势产业实现增加值36.3亿元，增长20.7%。中国纺织工业协会授予益阳“中国麻业名城”称号。新培育湖南名牌产品8个、国家免检产品3个。高新技术产业实现增加值8.1亿元，增长40%。农业产业化稳步推进。各类农产品加工企业达到3240家，其中市级龙头企业75家、省级龙头企业13家、国家级龙头企业2家；各类农民专业合作组织达到626个，经营收入达到56.4亿元，比上年增长20%。旅游业快速发展。共接待国内外游客407.9万人次，旅游总收入15.2亿元，比上年增长31.1%。

　　项目建设取得突破。重点项目建设进展顺利，重点调度的171个项目完成投资38.6亿元。益沅一级公路、桃江资江大桥竣工通车，惠同新材料二期、双龙橡塑、海纳百川、沅纸一期工程等产业项目竣工投产，益阳电厂二期工程顺利开工，赤山大桥、麻河口大桥、奎马公路、通乡通村公路、县城电网改造、城市垃圾处理工程、博云高科、惠同新材料三期、沅纸二期、柘溪电站扩容、修山电站、株溪口电站、东坪电站等一批重大项目正在抓紧建设。农村客运站场建设正式启动。在中心城区开始组织实施城市防洪续建工程、华港油脂等九大重点建设项目。项目前期工作进程加快，完成了319国道绕城高速公路、常吉高速公路安化连接线等项目的工可报告评审和风力发电资源普查、风电场选址及申报工作。项目规划工作得到加强，“十一五”规划全市330个重点建设项目中，有92个纳入省“十一五”规划“笼子”。小城镇建设迈出新的步伐，完成小城镇基础设施建设投资4.8亿元。

　　改革开放成效明显．全市累计完成351家国有企业的改革任务，占国有企业总数的91.9%。2005年共完成138家国有企业的改革任务，依法有偿理顺了19327名职工的劳动关系和8028名离退休人员的社会养老保险关系，获资产转让收入4.55亿元。农村综合配套改革顺利推进。政府机构改革、行政审批制度改革、事业单位人事制度改革、城建管理体制改革、粮食流通体制改革不断深入。对外开放水平进一步提高，招商引资年活动取得重大成果。组织参加了“中国湖南(香港)投资贸易洽谈周”活动，共签约项目27个，合同引资25.08亿元。全市直接利用外资3500万美元，比上年增长16.2%；实际利用内资85亿元，增长101.9%；出口总额7000万美元，增长44%。

　　社会事业全面发展．全市列入国家科技计划的项目5项、列入省级科技计划的项目17项。企业科技创新体系建设取得成效，科技进步对经济增长的贡献率达43%。教育事业协调发展，“普九”成果得到巩固，“两免一补”政策得到落实，教育资源整合工作取得重大进展。创建省级卫生城市顺利通过复检，“六大活动”(承办第十八至二十届世界杯羽毛球赛、举办益阳国际生态文化旅游论坛、举办中国第三届城市森林论坛暨创建国家森林城市、创建中国优秀旅游城市、国家卫生城市、国家环境保护模范城市)开始实施。公共卫生服务体系不断完善，农村医疗卫生条件得到改善，疾病预防控制工作落到实处，禽流感和霍乱防治取得胜利。食品药品监管工作得到加强。文艺创作取得较大成果，群众文化活动比较活跃，文化市场和出版物市场专项整治效果明显，市博物馆正式开馆。广播电视事业取得新的突破。成功承办了第十八届世界杯羽毛球赛。在全国第十届运动会上我市参赛运动员获得3枚金牌。群众体育和体育产业有了新的发展。农村部分计划生育家庭奖励扶助制度顺利推行，集中整治“两非” (非医学需要鉴定胎儿性别和选择性别终止妊娠)行动效果明显。环境监管和环境保护力度加大，城乡规划、国土和矿产资源管理不断加强。监察、审计、统计、新闻、人防、地震、档案、对台、外事侨务、地方志、民族宗教、无线电管理、残联、科协、老年科协、老龄等各项工作都有新的进步，国税、地税、金融、电业、工商、质量技术监督、烟草、石油、盐务、电信、移动、邮政、气象、水文、保险等中央、省驻益单位的工作都取得了新的成绩。

　　人民生活进一步改善。预计城镇居民人均可支配收入比上年增长8.2％，农民人均纯收入增长9.7％。年末居民储蓄存款余额为192.6亿元，比年初增长15.3％。就业再就业政策措施得到落实，规模进一步扩大。农村劳动力转移就业88.9万人，实现劳务收入50亿元。社会保险体系不断完善和巩固，企业离退休人员基本养老金按时足额发放，养老保险、城镇职工基本医疗保险、失业保险规范运行、稳步推进，工伤、生育保险全面启动。住房公积金制度建设取得新的成效。城市低保管理进一步规范，扶贫开发、五保供养、农村特困救助、城乡大病医疗救助等各项工作进一步加强，救灾救济工作有序开展，特困群体和受灾群众基本生活得到保障。

　　精神文明和民主法制建设得到加强。广泛开展了以邓小平理论、“三个代表”重要思想、科学发展观和社会主义和谐社会为主要内容的理论学习和教育活动。以纪念抗战胜利60周年为重点，大力弘扬了爱国主义和民族精神。深入学习贯彻《公民道德建设实施纲要》，公民思想道德素质不断提高，未成年人思想道德建设进一步加强。“十星”级农户和“五星”级居民户创建活动深入开展，文明城市、文明机关、文明村镇、文明社区创建活动扎实推进。各级政府认真贯彻执行本级人民代表大会及其常委会的决议、决定，自觉接受人大的法律监督、工作监督和人民政协及民主党派、工商联、无党派人士的民主监督，共办理市人大代表建议166件、市政协委员提案176件，办复率100%。依法进行了村委会第六次换届，进一步加强了社区建设，村民、居民自治和民主管理制度不断完善。“四五”普法成效明显，依法治市工作整体推进，制定了《益阳市全面推进依法行政五年规划》，行政执法和执法监督水平有新的提高。认真开展机关作风测评，政务公开工作全面推行，反腐倡廉、纠风治乱工作扎实推进，经济发展环境进一步改善。社会治安综合治理得到加强，严打整治斗争不断深化，信访和人民调解工作成效明显，安全生产工作取得成绩，社会大局保持稳定。全民国防教育继续深入开展，驻益人民解放军、武警部队和民兵预备役建设不断加强。工会、共青团、妇联等群众团体在三个文明建设中发挥了积极作用。

　　省市18件实事基本完成。省8件实事我市实施的21个项目全面完成。①新增城镇就业人员32768人，其中下岗失业人员再就业19582人，安排大龄就业困难对象再就业6031人。农村富余劳动力转移就业增加61829人。②改造和新建通乡公路160.6公里、通村公路504.2公里，列入洞庭湖畅通工程的桃江资江大桥建成通车。③企业基本养老保险新增参保人数18000人，失业保险新增参保人数9932人，医疗保险新增参保人数15411人，财政预算安排低保资金增长率达15%。④新建沼气池10216口。⑤完成农村中小学危房改造159所，17万平方米。⑥改造和新建乡镇卫生院52所。农村部分计划生育家庭奖励扶助资金发放及时到位率达100%。⑦城市污水综合处理率达到58.9%，生活垃圾无害化处理率达9.3%，城市建成区绿地率达31.9%，新建城镇经济实用房2.6万平方米。⑧改造乡镇敬老院20所，新增集中供养五保户899人。市政府承诺为民办理的10件实事基本完成。①完成无公害、绿色农产品认证81个，无公害、绿色农产品产地认定111.8万亩。②桃江资江大桥建成通车，茅草街港和白沙港千吨级码头建成投入使用。茅草街大桥的沱江长春桥、藕池河桥、南茅运河桥、五七运河桥及其接线路基全部完工，主桥因技术因素及资金较大幅度超概算，只完成了实物工程量的95%。③29所乡镇敬老院建设全部完成。④市中心医院门诊大楼主体工程已经竣工，10所农村卫生院建设全面竣工，桃江县新型农村合作医疗制度试点工作全面完成。@帮助10544名贫困人口越过了低收入线。⑥中心城区10个社区建设全面完工。⑦秀峰公园三期工程基本完成，益阳大道建设完成预定目标任务，完成了15条小街小巷的改造，高新区道路畅通工程建设基本完工。⑧天然气利用工程完成预定目标任务。⑨国有企业改革完成138家，新增城镇就业人员32768人。⑩中心城区户牌、街牌、路牌设置基本完成。

　　各位代表，刚刚过去的2005年，是经济社会快速发展、全面丰收的一年。成绩令人鼓舞，这是全市人民在中共益阳市委的正确领导下，负重自强、开拓进取、团结奋斗的结果；是各级各部门通力合作、协调配合、大力支持的结果。在此，我代表市人民政府，向全市人民和社会各界人士，向驻益人民解放军、武警部队、民兵预备役官兵和公安政法干警，向所有关心、支持益阳的改革与发展的朋友们表示衷心的感谢，并致以崇高的敬意！

　　回顾过去一年的工作，主要有以下五点体会：

　　一是必须坚持正确的发展思路，这是加快发展的根本。我们按照“坚持第一要务，突出两个重点，实施五大战略，加快‘三化’进程”的发展思路，聚精会神搞建设，一心一意谋发展，经济发展速度明显加快，经济效益明显提高，发展后劲明显增强，招商引资出现了前所未有的力度，项目建设出现了前所未有的强度，固定资产投资达到了前所未有的额度。

　　二是必须突出发展重点，这是加快发展的关键。加快益阳发展，实现后发赶超，必须突出重点，突破难点。在实践中，我们深刻认识到，要富民强市就必须加快推进“三化”进程，特别要推进工业化进程；推进“三化”进程就必须抓项目，特别要抓大项目；抓项目就必须招商引资，特别要引进战略投资者。

　　三是必须保持良好的精神状态和求真务实的工作作风，这是加快发展的保证。在困难和挑战面前，我们始终保持奋发向上的精神风貌，坚持自加压力，负重奋进，顽强拼搏，攻坚克难；始终发扬求真务实的工作作风，坚持脚踏实地，埋头苦干，艰苦奋斗，狠抓落实。

　　四是必须坚持以人为本、为民造福，这是政府工作的出发点和落脚点。我们的政府是人民的政府，为民造福是我们的神圣职责。我们始终恪守全心全意为人民服务的宗旨，自觉做到权为民所用、情为民所系、利为民所谋；始终把群众的冷暖、安危放在心上，认真解决事关民心、民情、民安、民乐、民忧的实际问题，真心实意为群众办实事、办好事，千方百计为群众排忧解难；始终坚持以富民的目标凝聚人心，以富民的举措激励人心，以富民的业绩鼓舞人心，努力实现好、维护好、发展好广大人民群众的根本利益。

　　五是必须搞好加快发展的“大合唱”，这是益阳的希望所在。2005年全市各项目标任务能够圆满完成，得益于全市上下实现了心齐、气顺、风正、劲足、同心同德干事业的大团结；得益于全市上下形成了想发展、议发展、谋发展、抓发展的合力；得益于全市上下强化了发展的责任意识、紧迫意识、目标意识、赶超意识、作为意识，唱响了加快发展的主旋律。

　　在充分肯定成绩的同时，我们必须清醒地认识到，与发达地区和周边市相比，差距很大，前进中面临的突出困难和问题是：经济总量仍然偏小；经济结构不够合理，增长方式比较粗放，传统产业改造升级任务艰巨；经济运行质量较低，财政特别是县乡财政十分困难；农业基础薄弱，农民持续增收困难；就业形势依然严峻，社会保障体系不健全，协调各种利益关系和改革攻坚的难度增加，影响社会稳定的因素仍然较多：计划生育工作滑坡；经济发展环境还不宽松，政府工作存在一些薄弱环节。我们必须高度重视，认真对待和解决这些问题。

　　二、“十一五”时期的奋斗目标和2006年工作安排

　　 （一）“十一五”时期的奋斗目标

　　“十一五”时期是贯彻落实科学发展观，加快建设小康益阳、生态益阳、平安益阳、诚信益阳、和谐益阳的重要时期，更是抢抓机遇、实现后发赶超的关键时期，我们必须乘势而上，加快发展。未来五年的奋斗目标是：

　　——总体目标。经济社会又快又好地发展，经济实力明显增强，进入全省第二方阵；中心城区快速发展，进入大城市的行列；各项社会事业全面进步，人民生活达到全省平均水平。

　　——经济发展。2010年，生产总值达到578亿元，年均增长12%，人均生产总值达到14400元，三次产业的比重调整为19：40：41，生产总值和人均生产总值提前3年在2000年基础上翻一番。财政总收入突破30亿元，年均增长15%以上。全社会固定资产投资达到249亿元，年均增长20%，5年累计完成投资890亿元。社会消费品零售总额达到204亿元，年均增长12%。城镇化率达到42%。

　　——社会事业。全面普及和巩固九年义务教育，高中阶段适龄人口入学率达到85%以上；全社会研究与开发经费占生产总值比重达到1.1%，科技进步对经济增长的贡献率达50%；执业医生达到9000人，病床达到9000张；新增城镇就业16万人，城镇登记失业率控制在5%以内；广播电视综合覆盖率达到98%。

　　——人民生活。农民人均纯收入年均增长8%，达到4370元；城镇居民人均可支配收入年均增长8.3%，达到12300元。

　　——可持续发展。全市总人口控制在472万人以内，年自然增长率控制在5‰以内；森林覆盖率达到50%，城市建成区绿化覆盖率达到38%，人均绿地面积达到9平方米；城市集中式饮用水源地水质达标率达到98%，农村饮用水源地水质达标率达到80%；工业固体废物综合利用率超过95%。

　　（二）2006年工作安排

　　2006年是“十一五”的开局之年，做好今年的工作，意义十分重大。当前和今后一个时期，我市既面临加快发展的良好机遇，也面临严峻的挑战。我们一定要抢抓机遇，沉着应对挑战，积极参与竞争，负重奋进，真抓实干，开创益阳科学发展的新局面。2006年工作的指导思想是：以邓小平理论和“三个代表”重要思想为指导，全面贯彻落实党的十六大和十六届五中全会精神，坚持以科学发展观统领经济社会发展全局，全力推进工业化进程，突出抓好招商引资和项目建设，切实加快社会主义新农村建设，大力提高城镇化水平，协调发展社会事业，努力实现经济社会又快又好的发展。主要预期目标是：生产总值增长11.5%；全社会固定资产投资增长20%；社会消费品零售总额增长12%；财政总收入增长15%；招商引资实际到位资金增长20%；城镇居民人均可支配收入增长8%，农民人均纯收入增长8%；新增城镇就业3.2万人，城镇登记失业率控制在5%以内，农村劳动力转移新增7万人；人口自然增长率控制在5‰以内。

　　1、突出实施工业强市战略，努力提高工业化水平

　　加快优势产业发展。围绕六大优势产业建设，进一步加大投入，加强指导，支持资水电力梯级开发、博云高科、泰格集团林浆纸一体化、惠同新材料三期、科力远二期、双龙橡塑、海纳百川、湘雅协和等一批重大产业项目的建设。坚持把发展高新技术产业作为提升我市工业化水平的战略举措来抓，充分利用“三基地一园区”的优势，大力发展壮大以新材料、新能源、生物医药、电子信息为主的高新技术产业。改造提升化工、冶金两大传统产业，建设以纳爱斯公司洗涤用品为主的精细化工基地，规范发展以安化、桃江为重点的矿产品采冶业。依托自身优势和高新技术，培育一批规模大、实力强、技术含量高的核心企业。

　　加快园区建设。努力构建高新区、区县(市)工业园、乡镇工业小区三级园区体系，进一步完善加快工业园区发展的政策措施，促进产业向工业园区集聚、企业向工业园区集中，使工业园区成为投资的洼地、招商的载体、企业的孵化器和经济的增长极。高新区、龙岭工业园、长春工业园要切实加大招商引资工作力度，引进更多的项目落户园区。沅江、南县、桃江、安化、大通湖等工业园区，要加快道路、供水、供电、通信等基础设施建设，壮大规模，办出特色。引导和鼓励乡镇工业企业向小区集中，做好“专、精、特、新”文章，加快发展乡镇工业小区。

　　推进企业技术进步。坚持依靠自主创新提升产业技术水平，着力形成以企业为主体、市场为导向、产学研相结合的技术创新体系。以突破行业关键技术和共性技术为重点，努力实施一批重点技术创新项目、重大产业技术开发项目和重大技术装备研制项目。坚持以信息化带动工业化，用高新技术和先进实用技术改造提升传统产业。大力支持粒粒晶米业、南洲酒业、建玲竹业、湘中木业、湘安钨业、湘诚实业、德盛纺织、东方水泥、洞庭宝胜珍珠、滨湖柴油机、宇晶机器等企业加快技改步伐，重点抓好一批投资过亿元的重大技改项目，全市完成技改投资22亿元。

　　大力发展民营经济。认真落实支持民营经济发展的政策。放宽市场准入，引导民营经济参与国有企业改革，进入金融服务、公用事业、基础设施建设等领域。拓宽民营经济投融资渠道，引导民营企业加强制度创新，促进民营经济产业层次和企业素质的提高。引导和支持中小型企业依靠科技和管理创新求发展，促进中小型企业总量上规模、结构上档次、质量上水平、管理上台阶。扶持壮大一批成长性好的“小巨人”企业。

　　2、巩固农业基础地位，加快社会主义新农村建设

　　切实抓好农业产业建设。突出发展特色农业。抓好优质种苗工程和农产品基地建设，提高农产品优质率。进一步完善“企业带基地连农户”的产业化运作模式，大力推进优质稻米、林纸板材、水产畜禽、棉麻纺织、油蔬茶果五大农业产业链建设。围绕农产品加工增值，着力扶持辐射范围广、带动能力强、有竞争实力的优质农产品加工龙头企业发展壮大。提高农业市场化程度。引导农民专业合作组织发展，大力培育农村经纪人，提高农民进入市场的组织化程度。加快推进农业标准化生产，健全农业技术推广、农产品市场、农产品质量安全和动植物病虫害防治体系。积极开展无公害、绿色、有机农产品认证和产地认定工作，提高农产品市场竞争力，把更多的农产品推向国际市场。

　　加强农村基础设施建设。继续抓好农田水利、农业机械化等项目建设。实施农村公路通畅和通达工程，加快乡村道路建设。发展农村通信。搞好村庄规划、建设和管理。抓好农村生产清洁工程、生活环境净化工程和生态文明提升工程建设，创建一批乡村清洁工程示范村。加快农村改水、改厨、改厕步伐，大力发展农村沼气，实现农民家园清洁、田园清洁、水源清洁。加快洞庭湖治理、山丘区水库除险保安等防洪工程建设，增强抵御洪涝干旱灾害的能力。做好防汛抗灾的各项准备工作，落实防汛抗灾责任制，确保安全度汛。

　　努力促进农民收入增长。大力实施科技入户工程、农村劳动力转移培训阳光工程，加强农民职业技能培训，增强农民致富本领。坚持“多予少取放活”的原则，切实落实免征农业税、粮食补贴、农机具购置补贴等一系列惠农富民政策，增加农民收入。进一步调整农村经济结构，发展农村二、三产业和多种经营，挖掘农业内部增收潜力。大力发展劳务经济，做好劳务输出的组织和服务工作，继续抓好三大劳务品牌(桃花江保姆、益阳厨师、安化保安)建设，切实保障进城务工农民的合法权益。整顿农资市场秩序，稳定农业生产资料价格。

　　加强农村基层组织建设。切实加强农村基层组织和基层政权建设，大力推进村务公开和村民自治，依法实行民主选举、民主决策、民主管理和民主监督。建立健全村民自治机制。加强村级班子队伍建设，优化结构，提高素质，落实报酬，提高基层组织的凝聚力和战斗力。

　　3、抓好招商引资和项目建设，增强持续发展的后劲

　　切实抓好招商引资。抓住国家实施“中部崛起”战略和沿海发达地区产业转移的重大机遇，进一步加大招商引资工作力度，以招商引资的大突破，促进经济社会的大发展，全市实现招商引资到位资金突破100亿元。突出招商引资重点。围绕六大优势产业、五大农业产业链和城乡基础设施建设，瞄准世界企业500强、国内企业500强和港澳台、长三角、珠三角地区招商引资，千方百计引进大项目，引进战略投资者。创新招商引资方式。坚持以商招商，提高服务质量，优化投资环境，确保引进项目的投资效益，以良好的投资效益吸引更多的投资项目；坚持以存量换增量，突出优势产业项目、资源性项目和国有企业的招商，实现以资源引项目；坚持以外资带内资，拉动民间投资，实现投资主体多元化；坚持政府招商与社会招商相结合，充分发挥委托招商、代理招商、亲情招商等方式的作用，形成政府引导、中介助力、园区带动、企业为主的招商体系。健全招商引资工作机制。强化招商引资目标管理，落实招商引资领导负责制，加强对区县(市)和市直单位的目标管理考核，不断完善合同引资、到位资金、项目履约率、项目贡献率“四位一体”的综合评价体系，严格考核，坚决奖罚兑现。认真组织参加首届中部地区(湖南)投资贸易洽谈会和第十届“厦交会”。

　　大力抓好项目建设。加快益阳电厂二期、柘溪电站扩容、修山电站、东坪电站、株溪口电站、沅茅二级公路、奎马二级公路、通村公路、茅草街大桥、东坪大桥、赤山大桥、麻河口大桥、华港油脂、太阳鸟新型游艇等在建项目的建设进度。认真做好益娄高速、319国道绕城高速、桃马公路、益阳至湘阴二级公路、南茅复线公路、202省道茅注段二级公路、益芦航道、金塘冲电站、白竹洲电站、风力发电、麻纺工业“两园一城”、安化矿产资源综合开发等项目的前期工作，力争一批项目在年内立项和开工建设。加强项目规划，争取更多的项目进入国家和省里的“笼子”。积极争取国家和省国债资金、专项拨款等各种资金，继续组织好银企洽谈，推进金融机构与各类投资主体之间的合作，认真落实城镇基础设施项目打捆申请开行贷款工作。加强项目管理，严格执行项目建设的各项制度，保证工程质量，提高投资效益。

　　4、深化改革开放，增强经济社会发展活力

　　继续抓好国有企业改革。按照三年完成国有企业改革任务的整体要求，突出重点，坚持成熟一家、改革一家的原则，积极推进国有企业改革。启动市属、区县(市)属大集体企业的改革。继续实行领导联系企业制度，强化企业改革指导组的作用，严格执行一个《决定》、两个《实施办法》、一个《补充意见》，坚持一个政策标准改到底。始终坚持以人为本，切实保障职工的合法权益，做到以改革促发展，以发展促稳定。加强对改革企业的指导、扶持和服务，确保改革一家，发展一家。

　　推进农村综合配套改革。以整合乡镇事业站所、减人、减事、减机构为重点，着眼于建立精干高效的农村基层行政管理体制，抓好乡镇机构改革；按照事权与财权相统一的原则，合理确定县乡财政体制；立足解决当前农村义务教育管理体制中投入保障偏低、资源配置不合理等突出问题，建立各级政府责任明确、财政分级投入、经费稳定增长、管理以县为主的农村义务教育体制。协调推进其他配套改革，稳定和完善以家庭承包经营为基础、统分结合的双层经营体制，完善农村土地流转、征用制度，探索和发展农业保险。加强农民负担监督管理，建立“农民自愿参与、村组自行组织、政府协调服务”的“一事一议”筹资筹劳新机制。

　　推进投融资体制等改革。探索完善市场主导、监管有力的投融资体制，培育规范投资中介服务组织，建立健全投融资市场，积极推行投资备案制和公益类项目代建制。加强政府对各类投资的监管服务。大力支持金融机构完善公司治理结构，健全内控机制，提高资产质量和服务水平，防范和化解金融风险。继续支持农村信用社的改革和发展。加强金融监管，维护金融稳定和安全。建立中小型企业信用体系，引导金融机构增加信贷投放，支持地方经济发展。加快要素市场和事业单位人事制度改革。

　　不断提高对外开放水平。支持企业扩大对外贸易。积极组织外贸企业参加各类出口产品交易会，支持外向型企业提高产品质量，优化出口商品结构，转变贸易增长方式，着力培育一批出口骨干企业和主导产品。鼓励各类企业扩大有效进口，积极引进国外先进技术、设备和管理经验，支持有条件的企业“走出去”，发展对外工程承包和劳务合作，促进产品和技术贸易交流。

　　提升服务业发展水平。发挥服务业吸纳就业、扩大消费的作用，进一步提升服务业发展层次。加快梓山湖、桃花江、南洞庭湖、柘溪库区等旅游区建设。抓好健身休闲游、山水生态游、历史文化和民俗风情游等功能产品的开发，促进旅游业快速发展。大力发展金融、保险、信息、咨询、评估和法律服务等现代服务业，加快发展连锁经营、物流配送、电子商务、特许经营等现代营销方式，扩大超市、仓储式商场等新兴物流业态，培育和引进竞争力较强的大型商贸物流企业，积极发展文化、体育、社区服务等需求潜力大的新兴服务业，运用现代经营方式和信息技术改造提升传统服务业。

　　5、着力推进城镇化进程，培育区域经济增长极

　　强化规划的引导和调控作用。完善城镇规划体系。完成《益阳市城市空间发展战略规划》和《益阳市城市总体规划(2004—2020)》的调整编制成果报批工作，加快控制性详细规划的编制，完成市规划展示大厅主体工程建设。加强对小城镇规划调整和修编工作的指导管理，启动农村村庄规划编制工作。加大规划执法力度，进一步完善规划管理制度，实行规划公示制，切实维护规划的权威。

　　加强城镇基础设施建设。切实加快中心城区建设。围绕创建国家森林城市、国家卫生城市、中国优秀旅游城市和国家环境保护模范城市，立足扩容提质、完善配套，抓好城市经营，加大城建资金归集力度，加快城市道路、供水、供电、供气、通信和垃圾、污水处理等基础设施建设。高标准完成益阳大道建设工程，完成五一东路下水道设施的配套改造。建好银城南路、团圆南路、杏花路、迎宾路、鹿角园西路、文昌路、罗溪路、秀峰东路和长春西路。建成梓山湖市民广场。基本完成会龙山公园改造工程和城市防洪续建工程目标任务。启动鹅羊池改造工程。做好江北污水处理厂建设项目的前期工作。积极引导房地产业健康发展，加快住宅小区建设，发展物业管理，提高人居质量。加快小城镇发展步伐。以健全功能和突出特色为重点，抓好县城和重点建制镇的建设，形成若干用地少、就业多、要素集聚能力强、人口分布合理的城镇群。

　　抓好城镇管理。大力推进城市管理行政执法体系建设，完善城市综合执法机构和相对集中行政处罚权制度，加大依法管理力度，建立长效管理机制，努力提升城市规范化管理水平。认真实施《益阳市土地储备管理办法》，高度垄断土地一级市场，严格依法处置城镇闲置土地。在中心城区推广使用预拌混凝土，提高建设工程质量。合理布局、规范发展集贸市场，取缔中心城区马路市场。完善户籍和流动人口管理办法，消除农民进城的障碍。深入开展创建文明卫生城镇活动，加强小城镇管理，促进镇容镇貌的改善。

　　6、切实加强财税工作，提高经济增长质量

　　着力培植财源。按照产业发展规划，大力加强财源建设，重点培育支柱财源，着力培植后续财源。进一步提高财政收入占生产总值的比重，提高税收收入占财政收入的比重，提高地方财政收入占财政总收入的比重。

　　切实加强征管。坚持依法征税，坚决制止越权减免税。依法勤征细管，严厉打击抗税、偷漏税、骗税行为，加大对易流失税源、小税种以及重点地区、重点行业、重点企业的征管力度，做到应收尽收。强化税务稽查，严禁买卖税收，严惩以税谋私行为。加强财税系统信息化建设，理顺征管秩序，改进征管手段，降低征管成本，提高征管效率，建立科学、规范、协调、高效的财税征管体制。

　　建立公共财政体系。继续深化部门预算、国库集中收付、政府采购和收支两条线管理制度改革。建立和完善公共财政体制，加大社会保障、新农村建设、公共服务、资源环境保护、自主创新和困难群众救助等领域的公共支出。严格支出管理和财政监督，强化节俭理财意识，压缩一般性支出。

　　7、大力推进社会事业，促进经济社会协调发展

　　优先发展教育事业。强化政府对义务教育的保障责任，巩固九年义务教育成果，大力推进素质教育。不断提高义务教育水平，认真落实“两免一补”政策，建立健全困难学生教育资助体系，基本消除九年义务教育阶段因贫辍学特别是女生辍学的问题。整合高中教育资源，加快普及高中阶段教育。支持高校扩大规模，提高办学质量。加快发展幼儿教育、职业教育和成人教育。支持和规范民办教育发展。积极推进农村中小学现代远程教育。落实教育督导评估制度。抓好教师培训工程，努力提高教师素质。

　　大力推进科技创新和人才工程建设。加大科技投入，促进科技工作由管理型向开发型转变，支持各类企业逐步建立研发机构，增强自主创新能力，提高科技竞争力。抓好能源、资源、环境、农业、信息等重大技术的开发利用。整合科技资源，做好科研项目和成果转化工作，加强科学普及，充分发挥科技对经济社会发展的引领和支撑作用。树立人才资源是第一资源的观念，建立人才评价、选拔任用、激励保障体制，激活人才市场，营造人才辈出、人尽其才的社会氛围。

　　努力提高公共卫生和医疗服务水平。建立健全疾病预防控制体系和突发公共卫生事件应急机制。扩大新型农村合作医疗制度试点范围，努力解决群众看病难、看病贵的问题。整顿和规范医疗市场秩序，加强医德医风建设。努力控制艾滋病、血吸虫病、乙型肝炎等重大传染病。加强卫生监督执法工作。加强食品、药品安全的监督管理。

　　切实加强人口和计划生育工作。以稳定低生育水平，提高人口素质为目标，夯实基层基础工作，突出抓好流动育龄妇女孕情监测、节育措施落实和对违法生育行为的处罚，加大打假治水和后进整改工作的力度，严格执行一票否决制度，落实领导责任，坚决扭转计划生育工作的被动局面。

　　发展和繁荣文化体育事业。努力扩大益阳广播电视节目的覆盖面，提高有线电视收视质量，继续实施广播电视“村村通”工程。繁荣文艺创作，加强非物质文化遗产的保护、开发和利用，实现文化资源城乡共享，促进文化市场和文化产业健康发展，丰富群众文化生活。加强文物保护和管理。进一步实施《全民健身计划纲要》，办好第十九届世界杯羽毛球赛，组团参加湖南省第十届运动会，争创优异成绩。

　　协调推进其他各项社会事业。抓好妇女、儿童发展规划的实施。重视发展残疾人事业。提高国防动员能力，增强全民国防意识，深入开展“双拥”活动，加强人民防空工作。切实做好民族、宗教、外事、侨务、对台、气象、水文、地震、档案、保密、老龄、地方志等工作，促进各项社会事业稳步协调发展。

　　8、切实关注民生，努力构建和谐社会

　　切实做好就业再就业工作。把就业再就业工作摆在经济社会发展的突出位置，强化目标管理和“一把手”负责制，认真落实积极的就业政策，完善市场导向就业机制，健全就业援助制度，千方百计增加就业岗位。抓好再就业培训，统筹做好城镇新增劳动力、高校毕业生和复员转业军人的就业安置工作，鼓励和支持自谋职业、自主创业。加强劳动保障监察执法，保护劳动者合法权益。

　　进一步做好社会保障工作。抓好社会保险的扩面、基金征缴和监督管理工作，完善社会保障体系。积极探索养老、失业、医疗、工伤、生育“五险合一”的统一征缴模式，推进“金保工程”建设。。大力发展社会福利、社会救济和社会互助事业，切实做好城镇低保工作。

　　大力发展循环经济。坚持开发节约并重、节约优先，按照减量化、再利用、资源化的原则，大力推进节能节水节地节材，推行清洁生产，发展循环经济。保护和合理利用资源，采取有效措施，限制资源粗放式利用，促进优质资源向优势企业集中，逐步建立再生资源回收利用体系，形成低投入、低消耗、低排放和高效率的节约型增长方式。依法淘汰落后工艺和设备，关闭破坏资源、污染环境和不具备安全生产条件的企业。加快安化高明循环经济工业区的发展，支持赫山沧水铺发展再生资源回收利用产业和黄泥湖利用电厂煤渣发展建材产业。

　　加大环境保护力度。坚持污染防治与生态环境保护并重，坚决改变先污染后治理、边治理边污染的作法，切实解决影响经济发展特别是危害人民健康的突出问题。在全市认真组织实施好以治理大气污染为重点的“蓝天工程”、以治理水污染为重点的“碧水工程”、以治理噪声污染为重点的材宁静工程”。强化对水源、土地、森林等自然生态资源的保护。

　　关心困难群众的生产生活。抓好救灾救济工作，认真实施《益阳市城乡特困家庭医疗救助制度实施办法》。高度重视城镇困难人口的生活问题，推进“双联”送温暖活动。认真解决城镇拆迁户的安置和生活问题。制定规划，抓好廉租房建设。完善农村贫困救助制度、“五保”制度，全面实施《益阳市农村居民最低生活保障制度实施办法》。因地制宜地实行整体推进的扶贫开发方式，探索实行易地扶贫，对丧失劳动能力的贫困人口建立救助制度。切实关心库区移民的生活，抓好库区开发，维护库区稳定。

　　9、加强社会主义民主法制建设，提高社会主义精神文明建设水平

　　加强社会主义民主法制建设。积极推进民主政治建设，自觉接受人大及其常委会的监督，坚决执行人大及其常委会的决议、决定；自觉接受政协的民主监督，广泛听取民主党派、工商联、无党派人士的意见；认真办理人大代表的建议、批评、意见和政协委员的提案，巩固和发展民主团结、生动活泼、安定和谐的政治局面。努力扩大基层民主，切实加强社区建设，完善村(居)民自治和企业民主管理制度。依法管理宗教事务。充分发挥工会、共青团、妇联等群众团体的桥梁和纽带作用。贯彻依法治市战略，加强政府法制工作，深入开展普法教育，提高全民的法制意识。

　　推进社会主义精神文明建设。深入开展邓小平理论和“三个代表”重要思想的学习，加强落实科学发展观、构建社会主义和谐社会的宣传，强化理想信念教育和思想政治工作。加强社会公德、家庭美德和职业道德教育，特别是青少年道德品质教育，在全社会倡导爱国守法、明礼诚信、团结友善、勤俭自强、敬业奉献的基本道德规范。深入开展各项文明创建活动，继续抓好创建“十星”级农户和“五星”级居民户的活动，倡导文明健康的生活方式，提高城乡文明程度。深入开展扫黄打非行动，大力整治网吧，净化文化市场和社会风气。坚持正确的舆论导向，营造负重奋进、加快发展的良好氛围。

　　坚决维护社会稳定，抓好安全生产。认真实社会治安综合治理一票否决制度，建立和完善治安防控体系。正确处理人民内部矛盾，完善信访工作责任制，健全社会矛盾纠纷调处机制。积极防范和依法严厉打击各类严重刑事犯罪和经济犯罪活动，依法严厉打击地下“六合彩”等赌博活动，坚决扫除各种社会丑恶现象。高度重视安全生产，严格落实安全生产行政首长负责制和责任追究制。开展经常性的安全现场检查，加大对重点企业、重点场所的专项整顿，坚决整治安全隐患，严厉打击非法生产，坚决防止重特大安全事故发生，保障人民生命财产安全。

　　10、坚持依法行政，加强政府自身建设

　　加快政府职能转变。致力建设施政科学、服务到位、行为规范的公共服务型政府。大力推进政企、政事、政资、政府与市场中介组织分开，增强社会服务功能，着力解决政府缺位、错位、越位的问题，政府应当承担的，做到责无旁贷；企业自主经营的，政府不承揽包办；能用市场办法解决的，就不用行政手段。进一步推进行政审批制度改革，坚持不懈地把优化经济发展环境作为“一号工程”来抓，严格落实优化经济发展环境七项制度，坚决查处干扰和破坏投资环境的行为。大力整治乱收费、乱罚款、乱摊派现象，保护投资者和经营者的合法权益。完善政务中心建设，进一步规范收费行为，提高服务质量和工作效率。

　　提高依法行政水平。认真贯彻执行《益阳市全面推进依法行政五年规划》。严格执行执法责任制和执法过错追究制。抓好规范性文件的清理，建立规范性文件的定期评估、评价和备案制度。加强统计执法，惩治虚报、瞒报等违法行为，提高统计质量。加强对政府工作人员特别是行政执法人员的教育、管理和监督，提高执法水平。健全行政监督机制，强化监察、审计等监督职能。

　　加大政务公开力度。切实抓好行政管理规范化建设，做到程序规范、政策公开、制度透明。完善重大问题集体决策制度和专家咨询、社会听证、新闻发布制度，建设阳光政府。全面推行政务公开，确保广大人民群众的知情权、参与权和监督权。依法规范招投标和征地拆迁行为，严厉打击暗箱操作等违法违规现象。

　　切实加强政风建设。强化行政责任，确保政令畅通，大力推行无为问责制和责任追究制。坚持求真务实，治理文山会海，改进文风会风，克服形式主义和官僚主义。全面贯彻《中华人民共和国公务员法》，建设政治合格、纪律严明、业务精通、作风过硬的公务员队伍。建立健全教育、制度、监督并重的惩治和预防腐败体系，大力纠正行业不正之风，严厉查处腐败案件。政府工作人员要深入基层，深入群众，认真倾听群众呼声，关心群众生活，着力解决人民群众最关心、最直接、最现实的利益问题。2006年为民办好10件实事：①改扩建10所农村中心卫生院；②改扩建20所农村敬老院；③帮助l万名农村贫困人口解决温饱和越过低收入线；④改扩建12座变电站，启动第三期农村电网改造；⑤抓好社保扩面，企业基本养老保险参保人数新增2万人，失业保险参保人数新增1.61万人，医疗保险参保人数新增3.5万人；⑥基本完成农村中小学现有危房改造任务；⑦抓好中心城区10个社区建设；⑧在中心城区改造10条小街小巷；⑨为高级职称人员作一次健康检查；⑩启动60个全面小康示范村建设。

　　各位代表，做好今年的工作，为“十一五”起好步、开好局，对于全面建设小康社会、构建和谐益阳具有重大意义。让我们紧密团结在以胡锦涛同志为总书记的党中央周围，高举邓小平理论和“三个代表”重要思想伟大旗帜，全面落实科学发展观，同心同德，真抓实干，开拓创新，奋力拼搏，为实现全市经济社会又快又好发展而努力奋斗！
